# Understanding the state of health policy and systems research in West Africa and capacity strengthening needs: scoping of peer-reviewed publications trends and patterns 1990–2015

**DOI:** 10.1186/s12961-017-0215-7

**Published:** 2017-07-12

**Authors:** Selina Defor, Aku Kwamie, Irene Akua Agyepong

**Affiliations:** Ghana Health Service, Research and Development Division, Accra, Ghana

## Abstract

**Background:**

The need for locally-driven, locally-generated evidence to guide health policy and systems decision-making and implementation in West Africa remains urgent. Thus, health policy and systems research (HPSR) is a field with great potential for addressing many of the sub-region’s intransigent health challenges. This paper presents an analysis of trends and patterns of peer-reviewed HPSR publications across the Economic Community of West African States (ECOWAS), to help understand trends and patterns of HPSR publication and the degree of involvement of West African researchers in HPSR evidence generation in the sub-region. Our goal was to use the findings to inform the development of a sub-regional strategy to strengthen HPSR and its use to inform development and improvement of health outcomes.

**Methods:**

A scoping review was conducted over a 25-year period from January 1990 to September 2015. Literature searches were conducted in English and French using Google Scholar, PubMed Central and Cairn.info.

**Results:**

A total of 258 articles were retrieved. Of these, 246 were statistically analysed, with 54% having West African lead authors. Two thirds of the papers originated from three out of the 15 countries of the ECOWAS, specifically Nigeria (28.86%), Burkina Faso (21.54%) and Ghana (17.07%). Most authors were based in academic institutions and participation of authors from ministries of health, hospitals and non-governmental organisations was limited. English was the predominant language for publication even for papers originating from Francophone West African countries. There has been a progressive increase in publications over the studied period.

**Conclusion:**

Despite progressive improvements over time, West Africa remains a weak sub-region in terms of peer-reviewed HPSR publications. Within the overall weakness, there is country-to-country variation. The fact that only a handful of countries accounted for nearly 70% of the total volume of publications in West Africa attests to the great disparities in individual, institutional and contextual capacities for HPSR evidence generation. Bridging the gap between lead institutions (universities and research centres) and the practice community (ministries, hospitals, non-governmental organisations) is indispensable for ensuring the practical use of HPSR evidence. There remains a major need for investments in HPSR capacity building in West Africa.

## Background

The pressing need to address weak and fragile health systems to achieve better health outcomes has been at the core of the global health agenda for over a decade. Nevertheless, achievement of the health-related Millennium Development Goals in West Africa has been greatly hampered by weak and poorly functioning health systems, among other challenges [1]. Health systems have been defined as comprising all actors, organisations, institutions and resources whose primary purpose is to improve health [2, 3]. Apart from this defining goal, the health system has other intrinsic goals, namely (1) to be responsive to the population it serves (determined by the manner and the environment within which people are treated), and (2) to ensure a fair distribution of the financial burden of paying for health.

Health systems have been described as complex adaptive systems, given the fact that they are constantly adjusting in dynamic and unpredictable ways to changes within the system itself and/or in the broader context within which they operate [4]. This dynamism underscores the fact that pre-defined health system strengthening blueprints risk becoming ineffective beyond a certain time, and thus calls for regular production of evidence to inform national and local health systems development. Additionally, several studies have also accentuated the fact that evidence emanating from research can improve health policy development in terms of identifying issues for the policy agenda, informing policy decision and evaluating policy outcomes, and ultimately orienting efforts at strengthening health systems [5–8]. Health systems strengthening has been globally recognised as being critical to improving health outcomes, but the knowledge-base to support this effort in low- and middle-income countries (LMICs) has been rather weak.

Health policy and systems research (HPSR) is defined as a field that seeks to understand and improve how societies organise themselves in achieving collective health goals, and how different actors interact in the policy and implementation process to contribute to policy outcomes [9]. HPSR addresses health system and policy questions that are not disease specific, but concern systems problems that have repercussions on the performance of the entire health system. HPSR addresses a wide range of questions, from health financing, governance and policy, to problems with structuring, planning, management, human resources, service delivery, referral and quality of care in the public and private sector. Naturally, HPSR is a multidisciplinary field that blends economics, sociology, anthropology, political science, public health and epidemiology to draw a comprehensive picture of how health systems respond and adapt to health policies and how health policies are shaped by health systems and the broader determinants of health; it has policy as its focus and thus promotes work that explicitly seeks to influence policy [10, 11]. It is worth noting that global interest and recognition of the importance of HPSR has been emphasised in several action-oriented reports and events in recent years [12–24]. According to some authors, commitment to HPSR and its application is reflected in the surge in international investment in the field in recent times [25]. Meanwhile, several other studies have repeatedly highlighted the persisting gap between high-income countries (HICs) and LMICs in terms of generation and use of HPSR [25–27]. This situation has caused some authors to question the ripple-effect of all global actions in support of the development of HPSR in LMICs [22, 27, 28].

West Africa, with an estimated population of approximately 350 million, comprises 15 countries (Benin, Burkina Faso, Cape Verde, Cote d’Ivoire, The Gambia, Ghana, Guinea, Guinea Bissau, Liberia, Mali, Nigeria, Niger, Sierra Leone, Senegal and Togo) all of which are classified as low or lower middle income [29].

The sub‐region is home to an immense diversity of people, in terms of cultures, languages and religion. The complexities of the sub-region are layered in traditional ethnic, religious and language diversity, which is further heightened by the colonial legacy of fragmentation of the sub‐region by official language into Anglophone, Francophone and Lusophone.

Compared to other regions, HPSR publications in West Africa have been woefully inadequate [26]. The weak health research production in the sub-region has been attributed not only to limited research capabilities and weak training capacities [30, 31], but also to weak capacities for resource mobilisation as well as limited capacity to develop research collaborations among countries within the sub-region [31, 32]. Despite having the potential to address most of the region’s intransigent health challenges, the status of HPSR in West Africa has not been systematically studied.

The present study therefore aimed to scope the landscape and describe patterns and trends of Anglophone and Francophone HPSR publications across the Economic Community of West African States (ECOWAS) over the period 1990 to 2015. Our specific objectives were to describe trends, institutions, individuals and networks conducting HPSR, and the degree of West African-led involvement of researchers in HPSR evidence generation in the sub-region. The goal was to use the findings to inform the development of strategies to strengthen HPSR capacity, conduct and use for health systems strengthening and health outcome improvement within ECOWAS, including the establishment of HPSR networks. This paper focuses on the analysis of institutions and trends, and we do not present the analysis of individuals and networks.

## Methods

Our methodology was a scoping review of peer-reviewed publications over the 25-year period from January 1990 to September 2015. The theoretical foundations underlying this approach were based on the six-stage methodological framework developed by Arskey and O’Malley, which defines a scoping review as a “*technique to map relevant literature in the field of interest*”. According to these authors, the scoping review is performed by (1) identifying the research question, (2) searching for relevant studies, (3) selecting studies, (4) charting the data, (5) collating, summarising and reporting the results, and (6) consulting with stakeholders to inform or validate the findings. The method is described as a knowledge synthesis approach that addresses an exploratory research question aimed at mapping evidence and gaps in research related to a given field through a systematic search, selection and synthesising of existing knowledge [33]. The review sought to identify and retrieve peer-reviewed HPSR publications on West Africa to understand patterns of Anglophone and Francophone HPSR publications, to identify individuals and institutions conducting HPSR, and to ascertain the degree of West African-led involvement of researchers in regional HPSR evidence generation. Thus, the scoping review was considered relevant for a study whose aim is to map the breadth rather than the depth of evidence in the field of HPSR in West Africa.

### Searching for and selecting relevant studies

Literature searches were conducted in English and French using Google Scholar, PubMed Central and Cairn.info. Inclusion criteria were peer-reviewed publications from work conducted in any of the 15 ECOWAS member states published in English or French, whose objectives and/or keywords explicitly included mention of health systems or one or more of the WHO health systems building blocks. Apart from Cape Verde and Guinea Bissau, which are Portuguese-speaking, the remaining 13 countries of ECOWAS are English or French speaking. However, we did not have the language expertise to review the Portuguese literature.

Search terms employed were initially limited to “health systems” AND “name of ECOWAS country”, “health policy systems research” AND “West Africa”, “health systems research” AND “West Africa” or “name of ECOWAS country” and “health policy” AND “name of ECOWAS country”. These were later expanded to include “health care financing” AND “West African country”, “health service delivery” AND “West African country”, “health leadership” OR “governance” AND “West African country”, “health information systems” AND “West African country”, “health care” AND “human resource” AND “West African country”. French search terms employed were: Prestations services + soin de santé + Afrique de l’Ouest/un pays de l’Afrique de l’Ouest, Accès + soin + Afrique de l’Ouest, Accès + soin + un pays de l’Afrique de l’Ouest, Personnels de santé + Afrique de l’Ouest/un pays de l’Afrique de l’Ouest, Politiques pharmaceutique + Afrique de l’Ouest/un pays de l’Afrique de l’Ouest, système d'information santé + Afrique de l’Ouest/un pays de l’Afrique de l’Ouest.

**Fig. 1:**
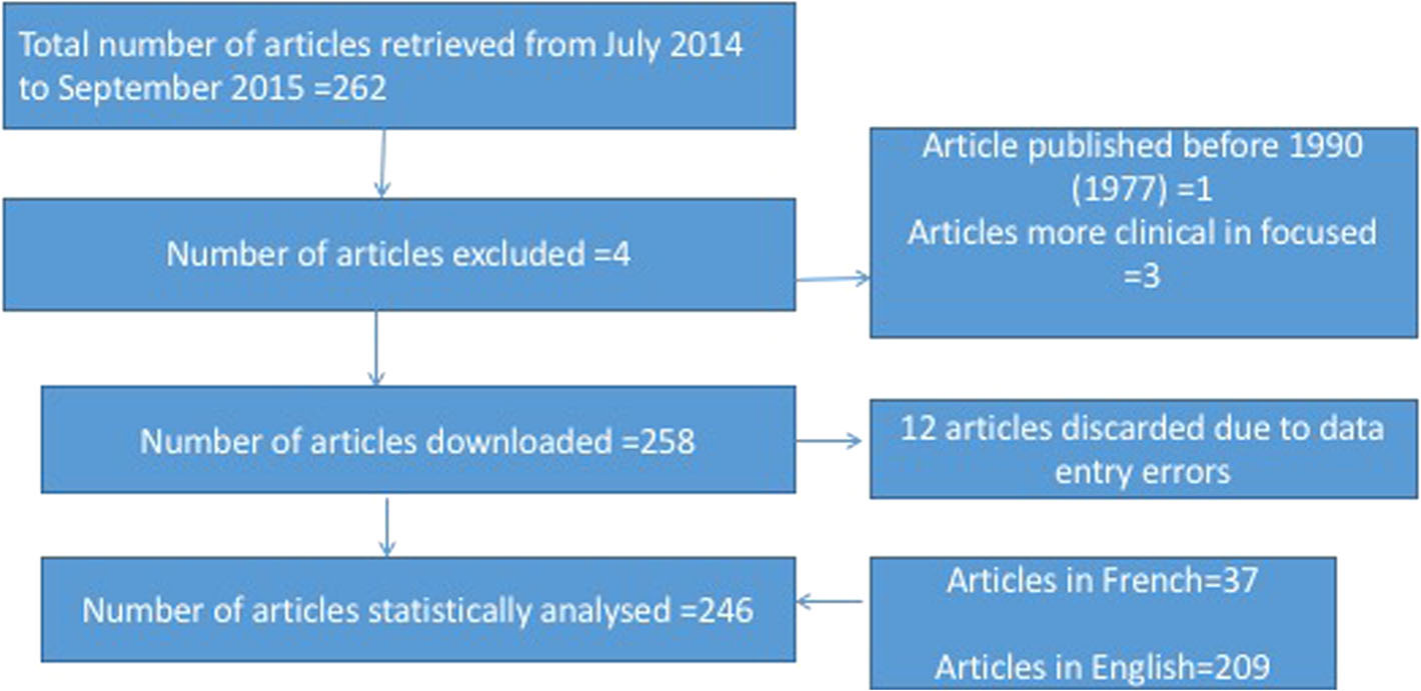
Flow diagram of inclusion

Figure 1 illustrates the flow diagram of inclusion of articles in the scoping review.

### Charting the data, collating, summarising and reporting the results

All eligible articles were downloaded and saved in an Endnote library. The data were compiled and imported in Microsoft Excel for validation and coding. From each publication included in the study, we extracted the following variables: language of publication, year of publication, study location, country in which the primary institution of the first author and co-authors were based, type of institution of the first author and co-authors (university, institute or research centre, hospital, non-governmental organisation or other), and number of West African first authors. We conducted some of the analysis in Excel and exported the data into STATA version 12 for further analysis.

### Consulting with stakeholders to inform or validate the findings

Consultation with stakeholders to validate the findings was performed at two sub-regional meetings. The first was a consultative ECOWAS health research strategic plan validation meeting of senior health researchers organised by the West African Health Organization (WAHO) in Abidjan in February 2015. Present at the meeting were senior researchers from universities, health research institutes and ministries of health across the ECOWAS sub-region. The second validation meeting was the inception meeting of the young West African health policy and systems researchers and implementers support network, known as the West African Network of Emerging Leaders organised in Accra in June 2015.

The search continued after the second validation meeting, from June to September 2015. The extension of the scoping and the addition of the WHO’s six health systems building blocks, and their sub-components, to define the scope of the review was due to the feedback from these two stakeholder validation meetings. The initial search, apart from stopping at 2014, had been narrowly limited to “health systems” OR “health policy” AND “name of ECOWAS country”. This narrow approach had yielded only 65 publications. In both validation workshops, respondents pointed out missing papers. To avoid bias, since not all possible researchers were members of these validation meetings, it was not considered advisable to add the papers they pointed out as missing, without making sure they met an objectively defined search inclusion criteria. The expansion of the search terms raised the number of papers to 262. It also dealt with most, but not necessarily all, of the missing papers indicated. However, for fairness in the comparative and trend analysis, we retained 246 papers, whilst acknowledging it is not an exhaustive list.

### Ethical considerations

This was a desk review of published literature already in the public domain. No primary data collection or analysis was carried out. There was therefore no need to seek ethical clearance or informed consent.

## Results

After reviewing the title, keywords and abstract of the papers retrieved, we included 246 articles from 14 out of the 15 West African countries in the analysis.

### Publication trends

**Fig. 2:**
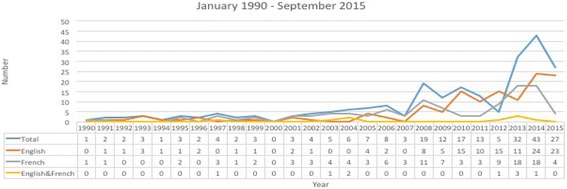
Trends in peer reviewed publications focused on Health Policy and Systems from West Africa

Figure 2 shows the total publications by year over the study period. The almost flat number of publications per year starts to show a slow rise in number of publications per year from 2001 onwards, which further accelerates from 2006 onwards.

### Publication by country location of the study

The observed growth in publications was due to the high volumes of publications from just a few countries. During the period reviewed, all the West African countries produced at least one HPSR-related article. Apart from Burkina Faso, which stands out considerably, the five Anglophone West African countries seemed to be publishing more than the majority of Francophone West African countries. Nigeria was the highest producing country, with a publication volume of 28.86% (*n* = 71) of the total regional production, followed by Burkina Faso (21.54%; *n* = 53) and Ghana (17.07%; *n* = 42). Indeed, Nigeria, Ghana and Burkina Faso alone accounted for more than two thirds of the articles retrieved.

When the publications over the period of review were related to the 2015 mid-year population, the difference between Anglophone and Francophone countries in terms of density of publications in relation to population was marked. Burkina Faso had the highest number of publications per million population. In this regard Nigeria, dropped close to the bottom of the list. Some of the smaller countries, such as Sierra Leone and The Gambia, showed a relatively high density (Table 1).

**Table 1:** Publications by country over the 25-year period per 1,000,000 of 2015 estimated population

2015 estimated mid-year	Population Popo	% 2015 pop	Publications	Per 1,000,000 of 2015 estimated population
Nigeria	183,523,432	53%	70	0.381
Ghana	26,984,328	8%	41	1.519
Côte d'Ivoire	21,295,284	6%	6	0.282
Niger	19,268,380	6%	6	0.311
Burkina Faso	17,914,625	5%	53	2.958
Mali	16,258,587	5%	11	0.677
Senegal	14,967,446	4%	9	0.601
Guinea	12,347,766	4%	3	0.243
Benin	10,879,828	3%	10	0.919
Togo	7,170,797	2%	1	0.139
Sierra Leone	6,318,575	2%	11	1.741
Liberia	4,503,439	1%	3	0.666
Gambia	1,970,081	1%	3	1.523
Guinea-Bissau	1,787,793	1%	1	0.559
Cape Verde	508,315	0%	0	0.000
Total Population WA	345,698,676	100%		0.000
Several West African			13	0.038
Several African			4	0.012
Multi International			1	0.003
			246	0.712

### Lead authorship and country of affiliation

Overall, 54% (*n* = 132) of the papers had a West African as the lead author. Table 2 summarises West African lead authors by country. However, there were many cases where, despite the lead author being West African, their primary affiliation was a non-West African institution, which accounted for the higher number of non-West African lead institutions compared to West African lead individuals. Generally, this was due to the lead authors being registered as PhD researchers in the Northern institution.

**Table 2:** West African Lead Authors by country in which research was conducted

Country	No. Papers	No with West African lead author	% with West African lead author
Benin	10	8	80%
Burkina Faso	53	14	26%
Cote d’Ivoire	6	5	83%
Gambia	3	1	33%
Ghana	42	31	74%
Guinea	3	0	0%
Guinea Bissau	1	0	0%
Liberia	2	0	0%
Mali	12	1	8%
Niger	5	3	60%
Nigeria	71	59	83%
Senegal	9	5	56%
Sierra Leone	11	0	0%
Togo	1	0	0%
Multi country West Africa	14	5	36%
Multi country Africa	2	0	0%
Multi country international	1	0	0%
Total	246	132	54%

### Institution of affiliation of lead author

Table 3 outlines details of the institution of affiliation of the lead author as well as the country in which the institute is located. The six most frequent institutions of affiliation of lead authors were University of Montreal (8.5%), University of Heidelberg (7.3%), University of Ghana and University of Nigeria (4.1% each), Johns Hopkins University (2.8%) and University of Columbia (2%).

**Table 3:** Details Institution of Lead author (detail)

Institution of lead author	Country of institution	Number	% of all institutions
SOUTHERN (WEST AFRICAN) UNIVERSITIES			
University of Ghana	Ghana	10	4.1%
University of Nigeria	Nigeria	10	4.1%
University of Calabar	Nigeria	4	1.6%
Nnamdi Azikiwe University	Nigeria	4	1.6%
University of Lagos	Nigeria	3	1.2%
University of Ibadan	Nigeria	3	1.2%
Ahmadu Bello University	Nigeria	2	0.8%
Cheikh Anta Diop University	Senegal	2	0.8%
Ebonyi State University	Nigeria	2	0.8%
L'Universite de Ouagadougou	Burkina Faso	1	0.4%
Olabisi Onabanjo University	Nigeria	2	0.8%
Nnamdi Azikiwe University	Nigeria	3	1.2%
Bayero University	Nigeria	1	0.4%
Ekiti State University	Nigeria	1	0.4%
Kwame Nkrumah University of Science and Technology	Ghana	1	0.4%
Niger Delta University	Nigeria	1	0.4%
ObafemiAwolowo University	Nigeria	1	0.4%
University of Abomey-Calavi	Benin	1	0.4%
Federal University of Agriculture	Nigeria	1	0.4%
University of Benin Teaching Hospital	Benin	1	0.4%
University of Cape Coast	Ghana	1	0.4%
University of Cocody	Cote d'Ivoire	2	0.8%
Delta State University	Nigeria	1	0.4%
University of Development studies	Ghana	1	0.4%
University of Ilorin	Nigeria	1	0.4%
University of Jos	Nigeria	1	0.4%
University of Sokoto, Nigeria	Nigeria	1	0.4%
		62	25.2%
NOTHERN UNIVERSITIES			
Universite de Montreal/University of Montreal	Canada	21	8.5%
University of Heidelberg	Germany	18	7.3%
John Hopkins University	USA	7	2.8%
Colombia University	USA	5	2.0%
London School of Hygiene & Tropical Medicine	UK	4	1.6%
The University of Western Ontario	Canada	4	1.6%
Norwegian University of Science and Technology	Norway	3	1.2%
University of Aberdeen	UK	3	1.2%
University of California	USA	3	1.2%
London School of Economics and Political Science	UK	2	0.8%
University of Ottawa	Canada	2	0.8%
University of Washington	USA	2	0.8%
King's College London	UK	2	0.8%
Radboud University Nijmegen	Netherlands	2	0.8%
Université Claude Bernard	France	2	0.8%
Liverpool School of Tropical Medicine	UK	2	0.8%
Ernst-Moritz-Arndt-Universität Greifswald (University of Greifswald)	Germany	2	0.8%
University of Illinois	USA	1	0.4%
Aarhus University	Denmark	1	0.4%
Institut d'etudes politiques de Grenoble	France	1	0.4%
Graduate Institute of International and Development Studies	Switzerland	1	0.4%
McGill University	Canada	1	0.4%
Université Catholique de Louvain	Belgium	1	0.4%
University of Alabama	USA	1	0.4%
University of Birmingham	UK	1	0.4%
University of North Carolina	USA	1	0.4%
University of Sydney	Australia	1	0.4%
University of Toronto	Canada	1	0.4%
University of Western Australia	Australia	1	0.4%
Hebrew University, Jerusalem	North	1	0.4%
Vrije Universitat Amsterdam	Netherlands	1	0.4%
Concordia University	Canada	1	0.4%
Yale School of Public Health	USA	1	0.4%
		100	40.7%
NORTHERN RESEARCH INSTITUTES AND GROUPS (NON UNIVERSITY)			
Institut français de recherche scientifique pour le développement en coopération	North	1	0.4%
Centre de Recherche Warocqué	Belgium	1	0.4%
Institute for Medical Technology Assessment	Netherlands	1	0.4%
Institute of Development Studies	UK	1	0.4%
Institute of Tropical Medicine-Antwerp	Belgium	4	1.6%
Memorial Sloan-Kettering Cancer Center	USA	1	0.4%
Royal Tropical Institute	Netherlands	1	0.4%
		10	4.1%
SOUTHERN RESEARCH INSTITUTES AND GROUPS (NON UNIVERSITY)			
Centre Muraz	Burkina Faso	6	2.4%
Navrongo Health Research Centre	Ghana	6	2.4%
Institut régional de santé publique	Benin	4	1.6%
Centre de Recherche en Santé de Nouna	Burkina Faso	2	0.8%
Laboratoire d'Etudes et de Recherche sur les Dynamiques Sociales et le Développement Local	Niger	2	0.8%
African Population and Health Research institute	Kenya	1	0.4%
Consortium de Recherche en Economie Sociale	Senegal	1	0.4%
Ecole Nationale Supérieure de Statistiques et d'Economie Appliquée	Cote d'Ivoire	1	0.4%
Institut de recherche en sciences de la santé	Burkina Faso	1	0.4%
Institut National de Recherches en Santé Publique	Mali	1	0.4%
Institut de Recherche en Science de la Santé	Burkina Faso	1	0.4%
Public Health Research & Development Centre	Gambia	1	0.4%
		27	11.0%
NORTHERN INSTITUTES - ALL OTHERS			
Center for Disease Control, Atlanta	USA	4	1.6%
Africa Development Bank	Multilateral	3	1.2%
Abt Associates	USA	3	1.2%
Astellas Pharma UK Ltd	UK	2	0.8%
ICF International	USA	2	0.8%
United Nations Children Emergency Fund	Multilateral	2	0.8%
Massachusetts General Hospital	USA	1	0.4%
German Development Cooperation	Germany	1	0.4%
United States Peace Corps	USA	1	0.4%
World Bank	Multilateral	1	0.4%
Institut de Veille sanitaire	France	1	0.4%
Family Care International	USA	1	0.4%
Helen Keller International	USA	1	0.4%
Save the Children	USA	1	0.4%
Surgeons OverSeas	USA	1	0.4%
University Research Co.	USA	1	0.4%
		26	10.6%
SOUTHERN INSTITUTES - ALL OTHERS			
Ghana Health Service	Ghana	4	1.6%
National Primary Health Care Development agency	Nigeria	2	0.8%
Agogo Presbyterian Hospital	Ghana	1	0.4%
Aminu Kano Teaching Hospital	Nigeria	1	0.4%
Federal Medical Centre	Nigeria	1	0.4%
Institut National de Santé Publique	Cote d'Ivoire	1	0.4%
Irrua Specialist Teaching Hospital	Nigeria	1	0.4%
Komfo Anokye Teaching Hospital	Ghana	1	0.4%
National Hospital	Nigeria	1	0.4%
Federal Ministry of Health, Abuja	Nigeria	1	0.4%
Ministère de la Santé et de l’Hygiène Publique	Cote d'Ivoire	1	0.4%
Ministère de la santé publique/Mission de coopération	Niger	1	0.4%
Burkinabe Public Health Association	Burkina Faso	1	0.4%
Cellule d’Analyse et de Prospective en Développement	Niger	1	0.4%
Partnership for Reviving Routine Immunisation in Northern-Maternal Newborn and Child Health	Nigeria	1	0.4%
Mediplan healthcare limited	Nigeria	1	0.4%
Nigeria Reinsurance Corporation	Nigeria	1	0.4%
		21	8.5%
Total		246	

Most lead authors (162/246) were affiliated with a university; 62% of these universities (100/162) were in the global North and 38% in West Africa. Northern universities were dominated by University of Montreal, which was the lead in 21 out of the 100 (21%) Northern institution leads, followed by University of Heidelberg (18/100). The West African universities were dominated by University of Ghana (10/63) and University of Nigeria (10/63).

When the Northern and the West African universities were combined in the analysis, the top five universities as affiliation of lead author were Montreal (14%), Heidelberg (11%), University of Ghana (6%), University of Nigeria (6%) and John Hopkins School of Public Health (4%).

Research institutes formed the next largest group of institutions (37/246). Most of these research institutes (27/37; 73%) were in West Africa, while the rest were in the North (Table 4), which is the reverse of the situation with universities. The Institute of Tropical Medicine in Antwerp was the institution of the lead author in 4 out of the 10 leader authors from Northern research institutes. Of the West African research institutes, the Navrongo Health Research Center in Ghana and the Centre de Recherche en Santé de Nouna in Burkina Faso led in publications, with six publications each. The Institut Régional de Santé Publique in Benin followed, with four publications. The culture of research institutions appears to be particularly strong in Ghana and Burkina Faso.

Universities alone accounted for more than two-thirds of the total publications, though the relevance of HPSR publication is dependent on its ability to influence policy and practice. It appears practitioners are less involved in the production of HPSR knowledge in West Africa. Stronger lead involvement of ministries of health and government agencies and even non-governmental organisations in HPSR evidence generation needs to be encouraged across the sub-region. There is much that other countries in the sub-region will be able to learn from Ghana in this regard (10 publications from the Ghana Health Service, with six from the Navrongo Health Research Center and four from other parts of the service). The Ghana publications from within the health service were generally led by Ghanaian researchers. Burkina Faso also has some lead in this area, despite the strong dominance of Northern researchers in the papers coming out of Burkina Faso.

**Table 4:** Summary of Institution of Lead author

Type of Institution	Location		
	Northern (% type of institution)	Southern (% type of institution)	Total (% of all institutes)
Government Agency	6 (40%)	9 (60%)	15 (6%)
Hospital	1 (14%)	6 (86%)	7 (3%)
International bilateral	1 (100%)	0 (0%)	1 (0.4%)
International Multi lateral	6 (100%)	0 (0%)	6 (2%)
NGO/Private	11 (73%)	4 (27%)	15 (6%)
Private (Other)	1 (33%)	2 (67%)	3 (1%)
Research Institute	10 (27%)	27 (73%)	37 (15%)
University	100 (62%)	62 (38%)	162 (66%)
Total	136 (55%)	110 (45%)	246 (100%)

### Language of publication

Even though French is widely spoken in the sub-region, most publications (84.96%) were in English, including publications from work performed in Francophone countries, and the one publication for Guinea Bissau, a Lusophone country.

## Discussion

This study confirms the observation of increased interest in HPSR globally as of 2008 [25]. Similarly, the publication rate within ECOWAS began accelerating significantly from 2008. This growth in peer-reviewed publications could be due to several factors. We speculate that perhaps the Mexico Summit in 2004, the first high-level ministerial discussion that placed health research back on the global health agenda, may have contributed. The series of global health research for a, including their action-oriented outcome documents and the activities leading to the Second Ministerial Forum in 2008, which was shortly followed by the First Global Symposium on Health Systems Research in 2010, may have all heightened the interest and recognition of the value of HPSR. A total of 33 articles were published between 1991 and 2003, the first 12 years preceding the 2004 Mexico Summit. The picture improved with a six-fold increase in publication over the next 12-year period, from 2004 to 2015, with a total of 212 publications.

Multiple global events and actions that emphasised the importance of health research for health systems strengthening for improved health outcomes may also have contributed. These are notably the Global Fora for Health Research and the Call to Action [14, 19, 20], the WHO Strategy on Research for Health [24], and the release of the United Kingdom DFID Research Strategy 2008–2013 [17], which emphasised the need to build research capacity for African countries to be able to identify their priority health needs and respond accordingly. Recognising the need to develop national level multi-disciplinary research capacity, the European Union made considerable investments in country-led research and knowledge generation efforts. With a budget of €6 billion under the Framework Programme 7, the European Union encouraged research collaborations with LMICs for cooperative health-research programmes and identified research on optimising healthcare delivery as one of the three priority areas [12].

Although HPSR publication output steadily increased in West Africa during the period reviewed, the volume remains minimal, and the longstanding gap between LMICs and HICs remains. With a publications volume of 212 from 2004 to 2015, West Africa is lagging far behind other regions. A similar HPSR stock-taking exercise conducted in the Eastern Mediterranean region over an 8-year period (2000–2008) showed a publication volume of 1489. The least producing country in this region (Yemen) produced just the same volume (*n* = 71) as the highest producing country (Nigeria; *n* = 71) in West Africa. A study that examined trends in international HPSR publications concluded that it will take more than 42 years for HPSR publications relative to the global South to reach the current rate of the global North at current publication rates [34]. Several factors may have accounted for this persisting gap in HPSR outputs in LMICs.

First, financial resource constraints, particularly a lack of national funding for health research, have been commonly identified as one of the major factors impeding HPSR productivity in LMICs [35] and holds true for West Africa. Despite all the international commitments to increasing domestic resources for health research, very few national resources are applied to any kind of health research in the sub-region, including HPSR [25]. A survey conducted by WAHO revealed that only one-third of the West African countries had put in place strategies to implement the international recommendation for the allocation of 2% of Ministry of Health budget and 5% of project budgets to research [31]. Most Ministry of Health budget documents audited had no budget line to support projects of the research for health apparatus within the Ministry of Health. Several studies have documented the fact that international multilateral and bilateral aid are the main funding source for HPSR through project grants in LMICs [36, 37]. However, LMIC institutions are less likely to receive core funding than institutions based in HICs [25]. External donor resources are a potential source of HPSR funding for ECOWAS member states, but unfortunately the capacity to develop strong proposals to compete for these funds is also weak in West Africa, limiting opportunities to tap into international competitive funding opportunities for HPSR [25].

Secondly, HPSR is context-specific and requires local actors with an understanding and appreciation of their own health systems challenges to drive the processes of evidence generation and application for health policy and systems, and ultimately health outcome improvement. This can only be the case on condition that the local actors have the requisite capacity. Capacity involves the ability of individuals, institutions and societies to perform functions, solve problems, and set and achieve objectives in a sustainable manner [38]. More specifically, HPSR capacity involves expertise and resources at the researcher, project and institutional levels to produce new knowledge and applications to improve the social response to health problems [39]. Regrettably, several studies have emphasised the weak and non-existing capacity for HPSR activities in LMICs [25]. The lack of capacity to produce HPSR in West Africa is clearly substantiated in this scoping review. The majority of the countries are producing very negligible amounts of HPSR publications, with the few who are producing a fair amount owing the volume of their publications to non-West African authors or West African lead author affiliation with institutions outside the region.

Third, apart from the weak individual and institutional capacities, the broader context within which the local researchers operate provides a disincentive to health research in general, including HPSR. A study carried out by WAHO also revealed that only 50% of West African countries have directorates within their ministries of health to oversee research for health, as well as strategic documents that outline health research priorities that include health systems-related issues [31]. Only five countries had national ethics committees with members trained in research ethics. Additionally, government health officials also lacked adequate capacity to support the translation of research findings into policy and practice, thus limiting the utility of the evidence generated. This further dwindled enthusiasm for the generation of more evidence.

Clearly, HPSR capacity needs strengthening in West Africa. There is scarce evidence on the most effective capacity strengthening initiative; however, various forms of in-country, cross-country, intra-regional and international collaborations have helped to strengthen research capacity and increased research productivity in several LMICs, including some of the stronger countries in West Africa, and the entire sub-region could learn from them. For example, the Medical Education Partnership Initiative in Nigeria, a consortium of six Nigerian universities working in partnership with two universities in the United States, built the research capacity of more than 1600 faculty, graduate students and resident doctors, between 2011 and 2013, through a train-the-trainers programme. The American partners train in-country resources persons, approximately six in each member university in nine different courses, who in turn replicated the workshops in their various institutions as part of a regular career development programme, using the same training materials. This improved capacity has not only led to increased number of publications in peer-reviewed journals but also increased responses to local and international grant applications and awards [40]. Additionally, collaboration between Thailand and South Africa with support from the United Kingdom moderately strengthened institutional capacity in South Africa, but led to significant individual research capacity strengthening. Capacity-building activities implemented during this collaboration included post-graduate training, joint proposal development, publication and dissemination of research results, staff secondment, mentorship and exchanges [41].

In the Southern Africa Development Community, a South–South collaboration between the region’s Center of Excellence for Biomedical Research and Training and Zimbabwe’s Blair Research Laboratory with support from the Danish Bilharziasis Laboratory helped build research capacities in Zimbabwe and other African institutions through doctoral-level training and joint research projects [42]. Finally, research capacity around social determinants of health was strengthened in several LMICs (Brazil, Colombia, Mexico, Kenya, South Africa and Tanzania) through a triangulation of South–North–South collaborative networks (SDH-NET) in partnership with Spain, Switzerland, the United Kingdom and Germany [43].

Several studies have also found a strong relationship between international research cooperation and increased scientific productivity [44–46]. They argued that the complexities involved in research require more specialised knowledge that no individual or a single country is expected to have. Thus, collaboration allows individuals to play to their strong suits, contributing their strongest skills and deepest knowledge, while relying on others to contribute other skills and knowledge [47]. At the macro-level, collaboration will enable institutions and countries to mobilise and use their differentiated capabilities to enhance the knowledge creation process towards increased productivity.

### Limitations of the study

Grey literature in a resource-poor setting can be very critical in determining outputs of research activities and knowledge production efforts. This scoping review was unfortunately limited to peer-reviewed publications, which means that a sizable volume of evidence to present a holistic picture of the situation may have been excluded. Additionally, publications in Portuguese, the third official language in West Africa, could not be retrieved for lack of language skills. Guinea Bissau is Portuguese speaking, but we found one English language publication from that country. No publications were found from Cape Verde. Given this language limitation, we cannot conclude there were no HPSR publications from Cape Verde or that the one English publication we found from Guinea Bissau is the only possible one. The field of HPSR is very broad and, despite trying to use a wide range of search terms, there is a possibility that we still missed some papers. The value of this study therefore lies in its comparative nature rather than in the absolute numbers. Finally, the study covered a period of 25 years and author institutional affiliations may have changed over this period.

## Conclusions

This first HPSR situational analysis in West Africa examined the general publication trends and sought to identify individuals and institutions involved in HPSR. The results showed a very slow but steady growth in HPSR publications since 2008, with an uneven distribution of output among countries and institutions. Nigerian and Ghanaian researchers produced more than 50% of the total regional HPSR publications. Francophone West Africa seem to have more non-West African researchers and institutions leading the HPSR agenda, and most Francophones published in English, making it the preferred language for scientific publication. HPSR research and publications in ECOWAS have been growing steadily over time. However, these publications are somewhat dominated by lead researchers affiliated with Northern institutions, especially in the Francophone and smaller Anglophone countries. Though the lead researchers usually have ECOWAS collaborators, it is important that the capacity to lead applied research such as HPSR is strengthened in ECOWAS.

Clearly, there is an urgent need for a critical mass of HPSR producers and consumers within the sub-region. These findings provide a fair idea of which West African institutions and individuals may eventually have the capacity for HPSR training and mentorship. Like many other LMICs, lack of resources (human and financial) has been identified as one of the factors limiting HPSR production in West Africa. Thus, for a resource-poor region, cross-country institutional collaborations that emphasise collaborative research agenda setting among researchers and research consumers within the sub-region is critical. Triangulating this collaboration with other Northern partners could further help address the identified challenges. Fortunately, there are some embryonic collaborative research activities within the sub-region that could be explored and further developed into a more sustainable South–South collaboration.

## Abbreviations

ECOWAS: Economic Community of West African States
HICs: high-income countries
HPSR: health policy and systems research
LMICs: low- and middle-income countries
WAHO: West Africa Health Organization.
